# Sir Peter J. Lachmann and Prof. Robert B. Sim: Gone but Will Never Be Forgotten!

**DOI:** 10.3390/v13050858

**Published:** 2021-05-08

**Authors:** Berhane Ghebrehiwet

**Affiliations:** Departments of Medicine and Pathology, Renaissance School of Medicine, Stony Brook University, Stony Brook, NY 11794, USA; Berhane.ghebrehiwet@stonybrookmedicine.edu

In addition to the ongoing COVID-19 pandemic, which has brought an unexpected global gloom, the beginning of this year was also particularly cruel to the complement field, as two of our greatest and dearest members—Sir Peter Lachmann and Prof. Bob Sim—passed away too soon. Although this dual loss has undoubtedly left a huge hole in our hearts, their memories will certainly live on forever with us as their fertile minds have collectively left a legacy of significant contributions to the immunology field in general and to the complement field in particular. More importantly, as outstanding mentors who have taught many researchers in the complement field, their presence will be felt forever, as their proteges will undoubtedly continue to work on the brilliant ideas that they left them with. Indeed, I have been one of the lucky individuals to have known both of these giants from both a personal and scientific angle, and therefore, they will forever be in my heart.

Sir Peter, or “Peter”, as he was affectionately called by many of his friends in the field, was a brilliant and omniscient man with a photographic memory that many of us envied and admired. When Peter got up to ask a question at the ICW meetings, the speaker was certain that he was going to get a question he could not answer but could learn from. The audience was, of course, always eager to hear what Peter has to say, as the question, almost invariably, would be impregnated with fine details including the names of the authors and the year the observation was made. In that sense, Peter was a human encyclopedia whose departure will leave a huge vacuum in the complement field.


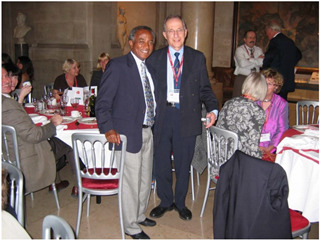


I was indeed one of the very lucky few who had the opportunity to work with Peter in his lab at Cambridge and see his genius at work. In the summer of 1982, I was invited to spend three months with Prof. Klaus Rother at the University of Heidelberg to test whether a degradation fragment from C3—which we named C3e—had the same activity as the leukocytosis mobilizing factor (LMF) described earlier by Profs. Ursula and Klaus Rother in Heidelberg. At that time, Peter’s lab was also working on the identification of the various degradation fragments of C3, including a fragment that he later called C3g, and therefore, he wanted to see whether these degradation fragments share any functional or structural similarity. After I finished my work in Heidelberg, I went to visit Peter in Cambridge. Once I arrived there, I had expected to stay in a hotel or campus accommodation. However, when Peter suggested and insisted that I stay in his house with his family, I was simply at a loss for words for this incredible generosity. In addition to being a brilliant man from whom I learned a lot, his humanity and unfiltered kindness are qualities that set Peter apart from others. In addition to working side-by-side with him in the lab, Peter also gave me the opportunity to know his extraordinary family—Lady Sylvia, an intellectual powerhouse in her own right, and their wonderful children, Helen, Robin and, Michael with whom I watched the World Cup of 1982. Although Peter and I were never able to publish the data from the work we conducted together in his lab, I still have “a Cambridge” folder with the data and correspondences from Peter, which I will keep as a treasure from a great man that I will fondly remember forever.

Bob Sim was another giant who left us too soon. Although, like many of my complement colleagues, I have known Bob through his work in the complement field and through the many ICW meetings we attended over the years, I also got a chance to know him better when my wife and I were given the opportunity to spend an NIH-funded sabbatical at the MRC Immunochemistry Unit in 1991–1992 and a Burroughs Welcome funded mini-sabbatical in 1995. The aim of the sabbatical was to clone the gene for C1q receptor. Since one of the major interests of the MRC Immunochemistry Unit led by Profs. Ken Reid and Bob Sim was also to unravel the structure of C1q and its binding partners or receptors, our sabbatical and collaboration was a perfect fit. Not surprisingly, the collaboration resulted in the identification of two unique receptors, in large part due to the incredible support and collegial atmosphere that both Ken and Bob provided. It would not be an exaggeration, therefore, to state that the MRC Immunochemistry Unit in those days was one of the best visited laboratories in the world, where young as well as seasoned researchers in the complement field from around the world would go to spend some time to perform collaborative research. In addition to being a center of excellence in complement research, the MRC Immunochemistry Unit was also popular with visiting researchers, because Ken and Bob made sure to provide an extraordinarily welcoming and nurturing atmosphere.

To lose two great scientists in one year is a great loss indeed. Thankfully, Sir Peter Lachmann and Prof. Bob Sim have left us with a rich collection of intellectual treasure through which their legacies will endure forever. Their loved spouses, Sylvia and Edith, and their children and grandchildren should know that the entire complement community is mourning with them. May they rest in peace.

